# Opioid use surrounding diagnosis and follow-up in patients with ankylosing spondylitis, psoriatic arthritis, and rheumatoid arthritis: Results from US claims databases

**DOI:** 10.1007/s10067-024-06945-0

**Published:** 2024-04-25

**Authors:** Anna Sheahan, Suzanne Anjohrin, Robert Suruki, Jeffrey L. Stark, Victor S. Sloan

**Affiliations:** 1 UCB Pharma, Smyrna, GA USA; 2 Sheng Consulting LLC, Flemington, NJ USA

**Keywords:** Ankylosing spondylitis, Medicaid, Opioids, Pain management, Psoriatic arthritis, Rheumatoid arthritis

## Abstract

**Objective:**

To describe patients’ use of opioids in the year preceding and year following new diagnosis of ankylosing spondylitis (AS), psoriatic arthritis (PsA), or rheumatoid arthritis (RA), compared with patients without the/se diseases.

**Methods:**

This study used US IBM^®^ MarketScan^®^ Commercial Claims and Encounters (CCAE) and Medicaid data and included three cohorts, comprised of incident cases of AS, PsA, or RA (2010–2017). Three matched comparator patients (without the incident disease) were selected for each patient within the disease cohort. Opioid use and appropriate treatment exposure (as defined by US guideline recommendations) in the 12-month baseline and follow-up periods were evaluated using descriptive analyses.

**Results:**

Prevalence of claims for opioids was higher for disease cohorts vs. comparators in CCAE; 36.4% of patients with AS, 29.5% with PsA, and 44.4% with RA did not have any claim for guideline-appropriate therapy in follow-up. Prevalence of claims for opioids was also higher for disease cohorts vs. comparators in Medicaid; 30.6% of patients with AS, 36.6% with PsA, and 65.4% with RA did not have any claim for guideline-appropriate therapy in follow-up.

**Conclusions:**

In patients with AS, PsA, or RA, there was high reliance on opioids at and around the time of diagnosis. Significant proportions of patients were not on appropriate treatment as defined by professional society post-diagnosis guidelines; this discordance between actual patient therapies and treatment recommendations may suggest a need for better awareness of appropriate pain management and treatment strategies in rheumatic diseases.

**Table:** 

**Key Points**
• *This study analysed opioid use among patients with ankylosing spondylitis (AS), psoriatic arthritis (PsA), or rheumatoid arthritis (RA), and adds to current knowledge by expanding beyond assessment of opioid use at diagnosis, to the year before and after diagnosis*. • *Opioid use was found to be highly prevalent in AS, PsA, and RA in the year prior to diagnosis and, interestingly, was still seen during the year after diagnosis*. • *Opioids are neither disease modifying, nor a targeted/recommended treatment for chronic autoimmune diseases. In addition to their association with significant economic costs, opioids are potentially hazardous and are not better than alternative treatments with superior safety profiles*. • *The reasons behind opioid prescribing patterns should be explored further to support movement to targeted therapies*.

**Supplementary Information:**

The online version contains supplementary material available at 10.1007/s10067-024-06945-0.

## Introduction

Ankylosing spondylitis (AS), psoriatic arthritis (PsA), and rheumatoid arthritis (RA)^1^ are chronic inflammatory diseases that affect the peripheral joints and/or axial skeleton, causing considerable pain and disability [1, 2]. Both inflammatory and non-inflammatory processes contribute to this pain [3, 4], therefore, therapies that reduce inflammation lead to improved clinical signs and symptoms, and slower disease progression [5–10]. Such therapies, including conventional synthetic and biologic disease-modifying antirheumatic drugs (csDMARDs/bDMARDs), are recommended by United States (US) treatment guidelines, with variations for each disease [11–13]. Opioids are generally not appropriate for the chronic treatment of these conditions and are not recommended by treatment guidelines [14]. However, chronic opioid therapy with continuous adherence monitoring is used to treat chronic non-cancer pain in select populations [15, 16].

Studies have identified concerning levels of opioid use among patients with rheumatic diseases [17–19]. In the US, patients with AS have high rates of chronic opioid use, especially among Medicaid patients [18]. A previous study investigating trends of opioid use in patients with RA in the US found a wide range in the percentage of rheumatology patients who received opioids (0–93%) [19]. Additionally, 40% of patients with RA had some or all of their opioid prescriptions ordered by a rheumatologist [19].

Opioid use among patients with AS has been associated with worse patient-reported outcomes including depression, Bath Ankylosing Spondylitis Disease Activity Index, and Bath Ankylosing Spondylitis Functional Index [20]. Long-term opioid use in patients with RA is correlated with diminished efficacy of csDMARDs and greater safety concerns [21]. Using opioids for pain management is connected with delayed initiation of appropriate treatment in patients with RA [17]. In patients with AS, opioid use is associated with higher initial treatment failure of tumor necrosis factor inhibitor therapy [22]. Thus, opioid use in rheumatic disease can lead to reduced efficacy of disease-targeted therapies and increased incidence of adverse events.

It is unknown when patients diagnosed with these diseases are initially exposed to opioids, and the patterns of opioid use surrounding diagnosis are not well characterized. A publication from Finland reported higher rates of opioid use in patients with PsA, RA, or axial spondyloarthritis compared with matched controls, with the highest rates in the months preceding diagnosis [23]. It is important to understand the time course of opioid use surrounding diagnosis to identify inappropriate use and areas for improving treatment approaches. Increased opioid use in these populations has individual and societal ramifications, including increased mortality rates, healthcare costs, needs for family assistance, and reduced productivity [24]. Therefore, it is important to assess opioid use surrounding diagnosis of these diseases to gain a better understanding of the temporal trends and associated risk factors.

This study describes patients’ opioid use in the years preceding and following new diagnosis of AS, PsA, or RA, compared with patients without these diseases.

## Materials and methods

### Study design and population

This was a retrospective cohort study of US IBM^®^ MarketScan^®^ Commercial Claims and Encounters (CCAE, with Medicare supplement) and Medicaid data. Analyses were conducted separately for CCAE and Medicaid; results were compared for each disease population in each database. IBM^®^ MarketScan^®^ is Health Insurance Portability and Accountability Act (HIPAA) compliant. Patient data were de-identified; thus, the use of the data does not constitute human subject research and does not require approval from an institutional review board. Analyses were performed with permission from the data owner to be presented at an aggregate level.

All patients were required to have ≥ 24 months preceding the incident diagnostic claim in which they were continuously enrolled and did not have a claim for the index disease. This 24-month pre-diagnostic period was used to select for newly diagnosed (incident) patients, and the 12 months directly preceding the index claim served as the baseline period. Patients were required to have 12 months of continuous enrollment following the index disease claim (follow-up period). Gaps of ≤60 days were allowed. The study design is shown in Fig. S1.

Each database population included three mutually exclusive cohorts comprising incident cases of AS, PsA, or RA that occurred between 2010–2017 among patients ≥ 18 years of age at the time of index claim. Supplementary Table S1 lists the International Classification of Diseases Ninth and Tenth Edition (ICD-9, ICD-10) codes used to identify patients for the specified cohorts. Patients with AS were eligible if their index claim had a rheumatologist provider or was an inpatient claim, or if they had ≥ 2 claims for AS by any provider > 7 days and < 360 days from the first in follow-up [25]. Similar definitions were used to determine eligibility of patients with PsA or RA. Patients with a cancer diagnosis (ICD-9: 140–209; ICD-10: C00–C96) in baseline or follow-up were excluded in the cohort and comparator groups due to the frequent opioid use in this population. Patients with rheumatic disease other than AS, PsA, or RA were not excluded from the comparator group.

Three comparator patients were randomly selected for each patient with disease, matched by age (year), sex, calendar year of index date (randomly selected date of an inpatient/outpatient claim that falls within the index year of the disease patient), region (CCAE only; US northeast, north central, south, west, or unknown), and insurance plan type (CCAE only; basic/major medical, comprehensive, exclusive provider organization, health maintenance organization, non-capitated/capitated point of service, preferred provider organization, consumer-driven health plan, or high deductible health plan). Comparator patients could be matched to more than one disease group, but within a specific group, they were only included once. Inclusion of a comparator group provides context for interpretation of prevalence in the disease cohorts, compared with the wider insured population.

Analysis was descriptive and focused on demographic and clinical characteristics, and treatment exposures of patients. Comorbidities of interest (depression, anxiety, fatigue, and fibromyalgia) were described in baseline and were based on the presence of ≥ 1 ICD-9 or ICD-10 claim for the specified comorbidity, except for fibromyalgia, which required ≥ 2 claims [26].

### Assessment of opioid use

Treatments for index disease were assessed separately in baseline and follow-up (inclusive). Opioid use was assessed by frequency (total and quarterly) and cumulative duration (chronic and long-term). The frequency (with 95% confidence intervals [CIs], using the asymptotic Wald test) of opioid use was assessed based on the presence of ≥ 1 pharmacy claim for opioids in the period of interest and was assessed quarterly in baseline and follow-up. Chronic opioid use was defined as a cumulative supply of ≥ 90 days in the period of interest.

Long-term use was assessed in follow-up and defined as ≥ 1 opioid claim in ≥ 3 quarters. Opioid claims occurring ≤ 7 days after a hospitalization or ≤ 2 days after an emergency room or urgent care center visit were not considered to minimize capture of opioid use for acute events like traumas. Prevalence ratios for opioid use were calculated by dividing the frequency of use among patients with AS, PsA, or RA by the frequency of use among matched comparators. History of opioid misuse was defined as ≥ 1 ICD-9 or ICD-10 claim in baseline for opioid misuse or abuse (≥ 1 ICD-9 code: 304.0, 305.5x; or ≥ 1 ICD-10 code: F11.10–F11.29).

Frequency of exposure to csDMARDs, bDMARDs, and nonsteroidal anti-inflammatory drugs (NSAIDs) was assessed based on the presence of ≥ 1 claim in the period of interest; there was also separate assessment by quarter in baseline and follow-up. Exposure to US guideline-recommended (appropriate) therapy, alone or with opioids, was assessed in baseline and follow-up. Exposure was based on the presence of ≥ 1 claim for appropriate therapy in the specified period of interest. Appropriate therapies by indication, including bDMARDs and NSAIDs for AS, and bDMARDs and csDMARDs for PsA and RA, are listed in Supplementary Table S3. In CCAE, treatment exposures were also considered separately in sub-analyses, stratified by biological sex and rheumatologist exposure in baseline or at diagnosis; these results are presented below and in Supplementary Table S2.

## Results

### CCAE population

**Fig. 1:**
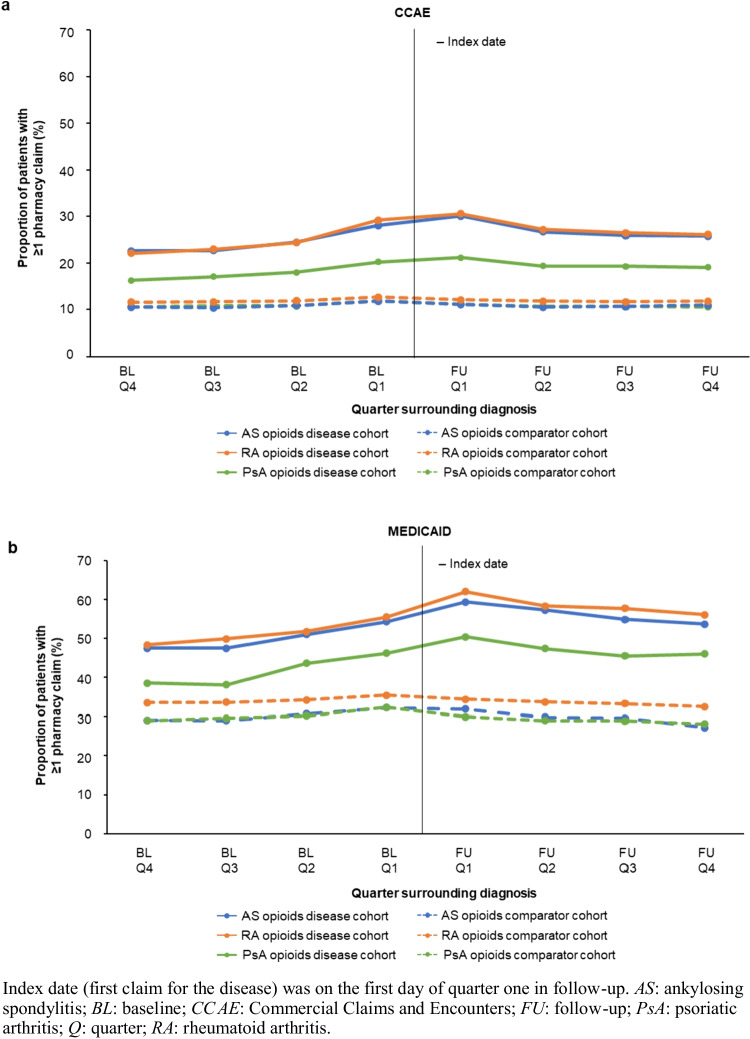
Proportion of pharmacy claims for an opioid by quarter in baseline and follow-up for AS, PsA, and RA in (**a**) CCAE and (**b**) Medicaid populations

The CCAE population included 5,769 patients with AS, 10,880 with PsA, and 91,722 with RA; Table 1 shows the patient characteristics of the disease and comparator cohorts. The proportion of patients with a rheumatologist visit at baseline or index were 42.4%, 45.2%, and 46.3% among AS, PsA, and RA patients, respectively (Supplementary Table S2). Baseline rates of depression, anxiety, fatigue, and fibromyalgia were higher in all disease cohorts vs. comparators. The frequency of NSAID and opioid use was higher for disease cohorts vs. comparators in baseline and follow-up, with NSAID and opioid use peaking in the quarter of diagnosis (Q1 of follow-up) for all disease cohorts (Fig. 1**, **Supplementary Fig. S2). Prevalence ratios for chronic opioid use in follow-up were higher for disease cohorts vs. comparators: 3.82 (95% CI: 3.51, 4.15) for AS, 2.41 (2.25, 2.58) for PsA, and 3.22 (3.15, 3.28) for RA. Prevalence ratios for long-term opioid use in follow-up followed a similar pattern: 3.51 (3.25, 3.79) for AS, 2.25 (2.11, 2.40) for PsA, and 2.99 (2.94, 3.05) for RA.

**Table 1:** Patient characteristics in 12-month baseline period for CCAE and Medicaid populations

	CCAE Population					
	AS		PsA		RA	
	Cases *N* = 5,769	Comparators^a^ *N* = 17,307	Cases *N* = 10,880	Comparators^a^ * N* = 32,640	Cases *N* = 91,722	Comparators^a^ *N* = 275,166
Age (years), mean (SD)	49.2 (15.0)	49.2 (15.0)	51.0 (12.5)	51.0 (12.5)	55.7 (14.4)	55.7 (14.4)
Male, n (%)	3,152 (54.6)	9,456 (54.6)	5,121 (47.1)	15,363 (47.1)	23,978 (26.1)	71,934 (26.1)
Female, n (%)	2,617 (45.4)	7,851 (45.4)	5,759 (52.9)	17,277 (52.9)	67,744 (73.9)	203,232 (73.9)
Region, n (%)						
Northeast	1,086 (18.8)	3,258 (18.8)	2,247 (20.7)	6,741 (20.7)	18,320 (20.0)	54,960 (20.0)
North Central	1,247 (21.6)	3,741 (21.6)	2,452 (22.5)	7,356 (22.5)	22,669 (24.7)	68,007 (24.7)
South	2,077 (36.0)	6,231 (36.0)	4,338 (39.9)	13,014 (39.9)	36,981 (40.3)	110,943 (40.3)
West	1,342 (23.3)	4,026 (23.3)	1,811 (16.6)	5,433 (16.6)	13,470 (14.7)	40,410 (14.7)
Unknown	17 (0.3)	51 (0.3)	32 (0.3)	96 (0.3)	282 (0.3)	846 (0.3)
Comorbidities, n (%)						
Depression	773 (13.4)	1,714 (9.9)	1,273 (11.7)	3,368 (10.3)	12,216 (13.3)	29,774 (10.8)
Anxiety	655 (11.4)	1,491 (8.6)	1,068 (9.8)	2,886 (8.8)	9,553 (10.4)	23,934 (8.7)
Psoriasis	68 (1.2)	135 (0.8)	5,748 (52.8)	297 (0.9)	944 (1.0)	2,402 (0.9)
Fatigue	1,040 (18.0)	1,860 (10.7)	1,647 (15.1)	3,710 (11.4)	20,907 (22.8)	33,315 (12.1)
Opioid abuse/misuse	56 (1.0)	79 (0.5)	61 (0.6)	98 (0.3)	592 (0.6)	750 (0.3)
Fibromyalgia	408 (7.1)	307 (1.8)	427 (3.9)	579 (1.8)	6,876 (7.5)	5,283 (1.9)
	Medicaid Population					
	AS		PsA		RA	
	Cases *N* = 337	Comparators^a^ *N* = 1,011	Cases *N* = 530	Comparators^a^ *N* = 1,590	Cases *N* = 7,369	Comparators^a^ *N* = 22,107
Age (years), mean (SD)	44.0 (12.7)	44.0 (12.7)	41.5 (11.7)	41.5 (11.7)	46.2 (12.0)	46.2 (12.0)
Male, n (%)	142 (42.1)	426 (42.1)	141 (26.6)	423 (26.6)	1,446 (19.6)	4,338 (19.6)
Female, n (%)	195 (57.9)	585 (57.9)	389 (73.4)	1,167 (73.4)	5,923 (80.4)	17,769 (80.4)
Comorbidities, n (%)						
Depression	145 (43.0)	337 (33.3)	194 (36.6)	543 (34.2)	3,015 (40.9)	7,921 (35.8)
Anxiety	115 (34.1)	274 (27.1)	173 (32.6)	436 (27.4)	2,517 (34.2)	6,105 (27.6)
Psoriasis	1 (0.3)	5 (0.5)	321 (60.6)	11 (0.7)	65 (0.9)	126 (0.6)
Fatigue	83 (24.6)	174 (17.2)	106 (20.0)	246 (15.5)	1,943 (26.4)	3,865 (17.5)
Opioid abuse/misuse	16 (4.7)	58 (5.7)	19 (3.6)	60 (3.8)	345 (4.7)	748 (3.4)
Fibromyalgia	36 (10.7)	31 (3.1)	56 (10.6)	50 (3.1)	956 (13.0)	914 (4.1)

*AS:* ankylosing spondylitis; *CCAE:* Commercial Claims and Encounters; *RA:* rheumatoid arthritis; *PsA:* psoriatic arthritis; *SD:* standard deviation. ^a^Comparator patients did not have AS, PsA, or RA diagnoses

Timing of first bDMARD use differed between disease cohorts. For AS, there was an increase in the frequency of bDMARD use from the quarter preceding diagnosis (5.8%) to the quarter of diagnosis (18.6%), with frequency plateauing at 19.1% through follow-up (Supplementary Fig. S2). For PsA, baseline frequency of bDMARD use was higher than the other disease cohorts (12.5–15.0%), likely due to pre-existing treatment of comorbid psoriasis (PSO). Use further increased in the quarter of diagnosis (31.0%), reaching 38.1% in the last quarter (Q4) of follow-up. Baseline frequency of bDMARD use for RA was 1.1–1.4%, with an increase during follow-up from 4.2% in the first quarter (Q1) to 9.7% in Q4.

**Fig. 2:**
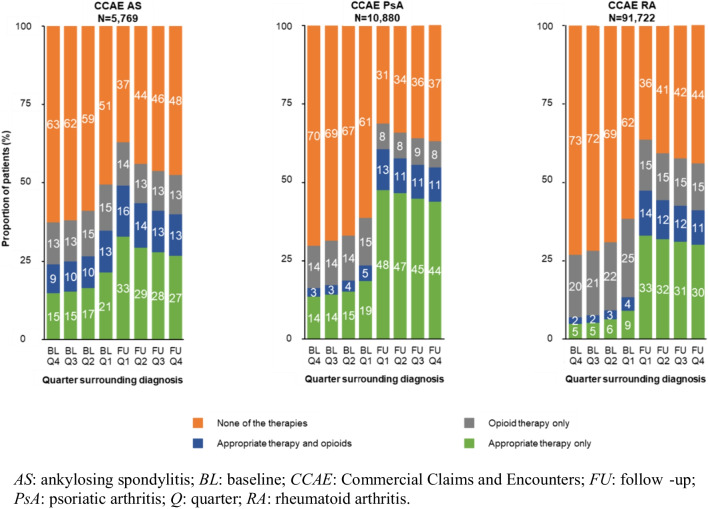
Proportion of disease appropriate therapy and opioids, alone or in combination, for disease by quarter in baseline and follow-up for AS, PsA, and RA in CCAE population

When considering exposure to appropriate therapy, 63.6% of the AS cohort, 70.5% of the PsA cohort, and 55.6% of the RA cohort had ≥ 1 claim for appropriate therapy during follow-up. The highest exposure to appropriate therapy occurred in Q1 of follow-up, at 49.2% for AS, 60.5% for PsA (66.9% and 53.2%, respectively, for patients with and without a diagnostic claim for PSO), and 47.4% for RA (Fig. 2). For AS, opioid monotherapy remained steady throughout follow-up at 12.5–13.8%, while 13.2–16.3% of patients received opioids with appropriate therapy. In Q4 of follow-up, only 40.0% of patients with AS had ≥ 1 claim for appropriate therapy. PsA showed the highest frequency of appropriate therapy at 54.6% in Q4 of follow-up, with opioid monotherapy in 8.4% and opioids with appropriate therapy in 10.8%. For RA, opioid monotherapy remained at 14.9–16.2% throughout follow-up, with 30.1–33.0% receiving appropriate therapy without opioids, and 11.2–14.4% receiving opioids with appropriate therapy.

#### Sub-analysis by biological sex

Frequency of opioid use in follow-up was similar in females vs. males for AS (45.1% vs. 43.2%) and RA (47.4% vs. 45.9%). For PsA, females were more likely to receive opioids than males (40.4% vs. 32.1%). Frequency of chronic opioid use in follow-up among females and males was 19.2% and 19.4% in AS, 13.5% and 10.6% in PsA, and 18.9% and 18.2% in RA, respectively. When considering comparators, prevalence ratios for long-term opioid use were similar across patient sexes among AS and RA cohorts: 3.70 for females with AS (95% CI: 3.30, 4.15) vs. 3.35 for males (3.02, 3.72); and 2.97 for females with RA (2.90, 3.03) vs. 3.07 for males (2.95, 3.19). The long-term opioid prevalence ratio in the PsA cohort was lower among males; 1.99 for males (1.80, 2.20) vs. 2.48 for females (2.27, 2.70).

#### Sub-analysis by rheumatologist exposure

The proportion of patients with opioid exposure by quarter was lower with rheumatologist exposure in the AS and RA cohorts; proportions did not differ for PsA (Supplementary Fig. S3). Females were more likely to have rheumatologist exposure at baseline or diagnosis (Supplementary Table S4).

Exposure to bDMARDs in follow-up was higher in patients with AS with rheumatologist exposure vs. no exposure, with less prominent differences in PsA and RA (Supplementary Fig. S3). Opioid monotherapy was lower in patients with rheumatologist exposure.

### Medicaid population

The Medicaid population included 337 patients with AS, 530 with PsA, and 7,369 with RA (Table 1). The mean age of the cohorts ranged from 41.5–46.2 years. Baseline rates of depression, anxiety, and fatigue were higher in the disease cohorts compared with comparators. Between 3.4% and 5.7% of patients in Medicaid had ≥ 1 ICD-9 or ICD-10 code for opioid abuse.

The frequency of NSAID and opioid use by quarter was higher for disease cohorts vs. comparators throughout follow-up, with frequency peaking in the quarter of diagnosis for all disease cohorts, except for NSAIDs in the AS cohort (Fig. 1, Supplementary Fig. S2). While the frequency of opioid claims dropped following diagnosis, the decrease was limited, with frequencies generally higher than those in baseline. Frequency of NSAID use was relatively stable among patients with AS after diagnosis; frequency in patients with PsA and RA was approximately 10% lower than the peak level by Q4 of follow-up. There was an increase in the frequency of bDMARD use for AS from Q4 of baseline (4.2%) to Q1 of follow-up (12.5%), and for PsA (11.7% to 27.4%). For RA, the increase was more gradual, with the highest frequency (4.9%) occurring in Q4 of follow-up.

Frequency of chronic opioid use in baseline was high, ranging from 21.8–25.4% in PsA- and RA-matched comparators, respectively, with higher frequencies in the disease cohorts, ranging from 31.3% in PsA to 45.7% in AS (Table 2). A similar pattern was seen throughout follow-up, with prevalence ratios demonstrating higher use in disease cohorts vs. comparators: 2.21 (95% CI: 1.90, 2.58) for AS, 1.82 (1.58, 2.10) for PsA, and 1.98 (1.92, 2.04) for RA. A similar trend was observed for long-term opioid use, with prevalence ratios of 2.05 (95% CI: 1.77, 2.39) for AS, 1.81 (1.58, 2.07) for PsA, and 1.93 (1.87, 1.99) for RA.

**Table 2:** Prevalence of treatment exposures over the 12-month baseline and follow-up periods surrounding diagnosis (index code) in CCAE and Medicaid populations

	CCAE Population					
	AS		PsA		RA	
	Cases *N* = 5,769	Comparators^a^ *N* = 17,307	Cases *N* = 10,880	Comparators^a^ *N* = 32,640	Cases *N* = 91,722	Comparators^a^ *N* = 275,166
Opioids						
Any in BL, n (%) [CI]	2,446 (42.4) [41.1–43.7]	4,187 (24.2) [23.6–24.8]	3,847 (35.4) [34.5–36.3]	7,949 (24.4) [23.9–24.8]	42,064 (45.9) [45.5–46.2]	71,097 (25.8) [25.7–26.0]
Any in FU, n (%) [CI]	2,541 (44.0) [42.8–45.3]	4,061 (23.5) [22.8–24.1]	3,975 (36.5) [35.6–37.4]	7,661 (23.5) [23.0–23.9]	43,094 (47.0) [46.7–47.3]	68,805 (25.0) [24.8–25.2]
Chronic in BL, n (%)	912 (15.8)	817 (4.7)	1,030 (9.5)	1,578 (4.8)	13,357 (14.6)	15,571 (5.7)
Chronic in FU, n (%)	1,114 (19.3)	875 (5.1)	1,322 (12.2)	1,648 (5.0)	17,179 (18.7)	16,010 (5.8)
Long-term in FU, n (%)	1,211 (21.0)	1,036 (6.0)	1,449 (13.3)	1,931 (5.9)	18,615 (20.3)	18,664 (6.8)
Other treatments, n (%)						
NSAIDs in BL	2,757 (47.8)	4,031 (23.3)	4,876 (44.8)	7,992 (24.5)	47,345 (51.6)	67,543 (24.5)
NSAIDs in FU	3,123 (54.1)	3,945 (22.8)	5,547 (51.0)	7,581 (23.2)	48,023 (52.4)	66,072 (24.0)
csDMARDs in BL	572 (9.9)	415 (2.4)	1,957 (18.0)	822 (2.5)	16,494 (18.0)	7,946 (2.9)
csDMARDs in FU	938 (16.3)	405 (2.3)	5,165 (47.5)	804 (2.5)	51,334 (56.0)	7,988 (2.9)
bDMARDs in BL	396 (6.9)	55 (0.3)	2,030 (18.7)	85 (0.3)	1,619 (1.8)	552 (0.2)
bDMARDs in FU	1,436 (24.9)	56 (0.3)	5,037 (46.3)	99 (0.3)	10,613 (11.6)	618 (0.2)
	Medicaid Population					
	AS		PsA		RA	
	Cases *N* = 337	Comparators^a^ *N* = 1,011	Cases *N* = 530	Comparators^a^ *N* = 1,590	Cases *N* = 7,369	Comparators^a^ *N* = 22,107
Opioids						
Any in BL, n (%) [CI]	222 (65.9) [60.5–70.9]	488 (48.3) [45.1–51.4]	329 (62.1) [57.8–66.2]	756 (47.5) [45.1–50.0]	5,273 (71.6) [70.5–72.6]	11,332 (51.3) [50.6–51.9]
Any in FU, n (%) [CI]	248 (73.6) [68.5–78.2]	453 (44.8) [41.7–47.9]	351 (66.2) [62.0–70.2]	738 (46.4) [43.9–48.9]	5,668 (76.9) [75.9–77.9]	11,025 (49.9) [49.2–50.5]
Chronic in BL, n (%)	154 (45.7)	234 (23.1)	166 (31.3)	346 (21.8)	3,055 (41.5)	5,625 (25.4)
Chronic in FU, n (%)	174 (51.6)	236 (23.3)	204 (38.5)	336 (21.1)	3,745 (50.8)	5,673 (25.7)
Long-term in FU, n (%)	171 (50.7)	250 (24.7)	217 (40.9)	360 (22.6)	3,891 (52.8)	6,053 (27.4)
Other treatments, n (%)						
NSAIDs in BL	198 (58.8)	453 (44.8)	321 (60.6)	737 (46.4)	4,937 (67.0)	10,688 (48.3)
NSAIDs in FU	213 (63.2)	444 (43.9)	356 (67.2)	755 (47.5)	5,269 (71.5)	10,430 (47.2)
csDMARDs in BL	17 (5.0)	21 (2.1)	119 (22.5)	30 (1.9)	931 (12.6)	446 (2.0)
csDMARDs in FU	36 (10.7)	16 (1.6)	245 (46.2)	32 (2.0)	2,586 (35.1)	477 (2.2)
bDMARDs in BL	18 (5.3)	0 (0.0)	85 (16.0)	2 (0.1)	93 (1.3)	47 (0.2)
bDMARDs in FU	58 (17.2)	1 (0.1)	211 (39.8)	0 (0.0)	468 (6.4)	53 (0.2)

*AS:* ankylosing spondylitis; *bDMARD:* biologic disease-modifying antirheumatic drug; *BL:* baseline; *CCAE:* Commercial Claims and Encounters; *CI:* 95% confidence interval; *csDMARD:* conventional synthetic disease-modifying antirheumatic drug; *FU:* follow-up; NSAID: non-steroidal anti-inflammatory drug; *PsA:* psoriatic arthritis; *RA:* rheumatoid arthritis. ^a^Comparators did not have AS, PsA, or RA diagnoses

In follow-up, 69.4% of patients with AS had ≥ 1 claim for appropriate therapy, ranging from 42.7–45.4% by quarter. Opioid use with appropriate therapy was more common than appropriate therapy alone; frequency of opioid monotherapy ranged between 24.9–33.2% by quarter. In PsA, 63.4% of patients had exposure to appropriate therapy in follow-up. By quarter, frequency of opioid use with appropriate therapy was 19.8–24.0%, opioid monotherapy was 25.1–26.6%, and appropriate therapy without opioids was 26.6–29.2%. Exposure to any appropriate therapy was lower in RA at 34.6%, ranging from 23.2–27.3% by quarter, with combination therapy in 13.1–15.9% of patients in each quarter and opioid monotherapy in 43.0–46.2% (Supplementary Fig. S4).

## Discussion

This analysis of US IBM® MarketScan® CCAE and Medicaid data demonstrated elevated chronic opioid use among patients with AS, PsA, or RA in the year surrounding diagnosis compared with matched comparators, adding to the current knowledge base by assessing beyond use at time of diagnosis.

High frequency of opioid use preceding AS, PsA, or RA diagnosis suggests that patients experience insufficient relief from their chronic pain, and the prolonged time to diagnosis, likely exacerbates this problem [27–29]. The probability of rheumatologist referral for patients with AS receiving opioid medications is significantly lower compared with patients receiving NSAIDs or csDMARDs [27]. Hypothetically, earlier diagnosis of disease could lead to less opioid use and increased uptake of appropriate therapy. However, frequencies of chronic opioid use in this study were higher in follow-up vs. baseline, contrasting previous reports that opioid purchases decreases after diagnosis of RA, undifferentiated arthritis, or Spondyloarthritis (SpA) [23]. Considering the differences between patients and comparators, patients with AS had the highest relative frequency of long term or chronic opioid use in follow-up, followed by patients with RA and then PsA. The prolonged delay from symptom onset to AS diagnosis compared with other rheumatic diseases [30] and the association of opioid use with reduced efficacy of AS-indicated therapies [22] may account for higher long-term or chronic opioid use among patients with AS. The observed lack of opioid discontinuation after diagnosis could represent a slow transition to appropriate therapies and/or the challenges associated with the discontinuation of opioids. A study of patients from Finland similarly observed an increase in opioid purchases among all inflammatory arthritides vs. comparators, with the largest odds ratio (OR) reported among patients with SpA (OR = 6.7) [23].

The PsA cohort had the highest increase in appropriate treatment following diagnosis. Notably, the PsA cohort had a high percentage of patients on bDMARD therapy in baseline, likely due to treatment for antecedent PSO. Overall, there was a trend toward decreasing exposure to appropriate therapy by quarter in follow-up, due to decreases in NSAID/csDMARD use, although use of bDMARDs increased or remained stable over time across all cohorts. These findings suggest that patients are still experiencing pain, and the peak in opioid use leading up to diagnosis suggests that this pain may be due to these diseases.

Across indications, patients with rheumatologist exposure at baseline or diagnosis had lower opioid monotherapy and higher bDMARD use in follow-up, which somewhat contradicts prior research proposing a link between rheumatologist exposure and opioid use [19]. Further research is required to determine whether the higher frequencies of bDMARD use were due to more severe disease in patients referred to rheumatologists, or reflections of rheumatologists having a more comprehensive understanding of disease-specific treatment guidelines than other healthcare professionals.

Although frequencies of opioid exposure were higher in the Medicaid disease cohort, this was also true for their comparators; therefore, the prevalence ratios for long-term and chronic use were lower in Medicaid vs. CCAE. These results are consistent with findings from the Medicaid and Children’s Health Insurance Program (CHIP) Payment and Access Commission which reported that Medicaid enrollees aged 18–64 had higher rates of opioid use than privately insured individuals [31]. Opioid monotherapy is problematic as it does not address the underlying cause of pain, results in inappropriately-managed patients, and puts patients at risk of other negative outcomes associated with opioid use.

### Strengths and limitations

This analysis represents a geographically expansive inquiry into opioid use in the US among patients with AS, PsA, or RA, permitting generalizability to larger disease-impacted populations across the country. This is further strengthened by using two representative populations in the study design. However, further studies are needed to determine whether a causal link between disease and opioid use exists.

This study has limitations that should be considered when interpreting the results. Matched comparators made the analysis more robust against potential confounders; however, as with most real-world data analyses, it is likely that there was some residual bias not fully addressed. Requiring only one ICD code for identification of AS, PsA, or RA patients may have led to the inclusion of misclassified patients, however, this method for classification aligns with previously validated algorithms [25] and is necessary to capture the time surrounding diagnosis. Although a more stringent inclusion criterion, such as requiring ≥ 2 diagnoses for the index disease 30 days apart, or restricting the ICD codes used, could further reduce the likelihood of misclassification, a more specific algorithm would likely impact how well patients on their diagnostic journey are captured; patients who are not properly diagnosed have the greatest risk for not being treated in accordance with guidelines and consequently may be inappropriately treated with opioids. Future studies may wish to investigate whether any meaningful differences in treatment patterns arise when more conservative definitions are employed.

Ideally, disease severity and time from symptom onset would be considered when examining treatment patterns; however, it is challenging to determine either of these reliably from claims data. Therapy exposure was based on the date of the claim, but the quantity or dose of medication covered by claim was not considered. It is possible that claims within a single quarter would have a supply extending into another quarter, leading to some misclassification of patients as unexposed in the later quarter (assuming there was no other claim). Additionally, it is possible that some patients took over-the-counter NSAIDs; despite the potentially lower out-of-pocket cost of prescribed NSAIDs. Inaccurate documentation of over-the-counter medication, not captured in claims data, use has been demonstrated in prior research [32].

The high frequency of opioid use identified in this study among patients with AS, PsA, or RA, both at and surrounding diagnosis, suggests the need for safer and more effective strategies for pain management in the treatment of these diseases. These strategies depend on early diagnosis and improved awareness of current guidelines among healthcare professionals. In 2017, a public health emergency was declared in the US to address the opioid crisis [33]. Changes were also made to rheumatic disease management such as implementing a treat-to target approach [34] and restricting initial opioid prescriptions to ≤ 7 days [35]. The combination of increased public, clinician, and government attention to the opioid crisis, and better targeted therapeutic options will likely lead to the continuation/extension of the existing decrease in opioid prescriptions and reduced disease burden in patients with AS, PsA, and RA. Future research questions include determining how to improve the diagnostic process, avoid unnecessary opioid exposure, and facilitate connection to appropriate treatments. This information could be used to guide patients and providers to appropriate therapies, reducing the individual, societal, and economic burdens of opioid abuse.

## Conclusions

These findings indicate that opioid use for management of pain associated with AS, PsA, or RA is highly prevalent in the US and continues after diagnosis. Considering prior research, these results have clear implications for the health of patients with AS, PsA, or RA. Reliance on opioids for pain management has been associated with substantial societal costs such as diversion, overdose, and addiction [36]. Opioids are not recommended for chronic use, nor as treatment for inflammatory arthritides.

Nonetheless, opioid addiction is pervasive; 8–12% of patients using opioids to manage chronic pain develop an addiction [37]. There is evidence that long-term opioid use is associated with lower efficacy in relieving pain compared to short-term use [21], while the risks of long-term use include the development of psychological addiction and/or abuse [38]. Combined with the significant socioeconomic costs, these considerations advocate a different approach to treatment of pain in inflammatory arthritides, centered on the use of evidence-based appropriate therapies. Better diagnosis, education, and adherence to guidelines may lead to improved treatment strategies and reduced use of chronic opioids to manage pain in rheumatic disease.

### Supplementary Information

Below is the link to the electronic supplementary material.

Supplementary file1 (DOCX 585 KB)

## Data Availability

Data from non-interventional studies are outside of UCB’s data sharing policy.

## Footnotes

1 These diseases are commonly reported in order of prevalence, however, in this manuscript they are presented alphabetically.
