# Supplementary material for: Opioid use surrounding diagnosis and follow-up in patients with ankylosing spondylitis, psoriatic arthritis, and rheumatoid arthritis: Results from US claims databases
Source: Clin Rheumatol. 2024 Apr 25;43(6):1897–907. doi: 10.1007/s10067-024-06945-0 (PMC11111565; doi:10.1007/s10067-024-06945-0)
Supplement: Supplementary file 1 — Supplementary file1 (DOCX 585 KB) [file 10067_2024_6945_MOESM1_ESM.docx]

Opioid use surrounding diagnosis and follow-up in patients with ankylosing spondylitis, psoriatic arthritis, and rheumatoid arthritis: Results from US claims databases

Anna Sheahan, PhD^1^, Suzanne Anjohrin, MPH^1^, Robert Suruki, ScD^1^, Jeffrey L. Stark, MD^1^, Victor S. Sloan, MD^2^

^1^UCB Pharma, Smyrna, GA, USA; ^2^Sheng Consulting LLC, Flemington, NJ, USA

**Correspondence to:**
Jeffrey L. Stark, MD, Head of Immunology Medical Affairs, UCB Pharma
Email: jeffrey.stark@ucb.com

**Journal of Clinical Rheumatology**

SUPPLEMENTARY MATERIALS

**Supplementary Table S1** ICD-9 and ICD-10 diagnosis codes for disease cohorts

| **Disease cohort** | **Diagnosis codes included in definition** |
| --- | --- |
| AS | ICD-9: 720.0  ICD-10: M45.X |
| PsA | ICD-9: 696.0  ICD-10: L40.50; L40.51; L40.52; L40.53; L40.59 |
| RA | ICD-9: 714.0; 714.1; 714.2; 714.81; 714.89; 714.9  ICD-10: M05.X; M06.X |

AS: ankylosing spondylitis; ICD: International Classification of Diseases; PsA: psoriatic arthritis; RA: rheumatoid arthritis

**Supplementary Table S2** Patients in CCAE population with rheumatologist visit in the 12‑month baseline period or at index date

| **n (%)** | **AS N=5,769** | **PsA N=10,880** | **RA N=91,722** |
| --- | --- | --- | --- |
| Rheumatologist visit in baseline period or index date | 2,444 (42.4) | 4,922 (45.2) | 42,430 (46.3) |

AS: ankylosing spondylitis; CCAE: Commercial Claims and Encounters; PsA: psoriatic arthritis; RA: rheumatoid arthritis

**Supplementary Table S3** Appropriate therapies by disease indication

| **Disease indication** | **Drug class^a^** | **Drugs included** |
| --- | --- | --- |
| AS | NSAIDs | All NSAIDs |
|  | bDMARDs | Adalimumab, certolizumab pegol, etanercept, golimumab, infliximab, secukinumab |
| PsA | csDMARDs | Cyclosporine, leflunomide, methotrexate, sulfasalazine |
|  | bDMARDs | Abatacept, adalimumab, apremilast, certolizumab pegol, etanercept, golimumab, infliximab, ixekizumab, secukinumab, ustekinumab |
|  | JAK inhibitors | Tofacitinib |
| RA | csDMARDs | Hydroxychloroquine, leflunomide methotrexate, sulfasalazine |
|  | bDMARDs | Abatacept, adalimumab, anakinra, certolizumab pegol, etanercept, golimumab, infliximab, rituximab, sarilumab, tocilizumab |
|  | JAK inhibitors | Tofacitinib |

AS: ankylosing spondylitis; bDMARD: biologic disease‑modifying anti-rheumatic drug; csDMARD: conventional synthetic disease-modifying antirheumatic drug; NSAID: non-steroidal anti‑inflammatory drug; PsA: psoriatic arthritis; RA: rheumatoid arthritis. ^a^Appropriate therapies as defined by current US guidelines for disease management. Originator therapies were not included due to limited uptake at the time of this study

**Supplementary Table S4** Patients in CCAE population with rheumatologist exposure over 12‑month baseline and follow-up periods, stratified by patient gender and age

|  | **AS N=5,769** | | | **PsA N=10,880** | | **RA N=91,722** | |
| --- | --- | --- | --- | --- | --- | --- | --- |
|  | **Rheum** | | **No rheum** | **Rheum** | **No rheum** | **Rheum** | **No rheum** |
| Age (yrs), mean (SD) | 46 (14.3) | 52 (14.9) | | 50 (11.8) | 52 (12.9) | 53 (13.4) | 58 (14.9) |
| Female, n (%) | 1,202  (49.2) | 1,415  (42.6) | | 2,759  (56.1) | 3,000  (50.4) | 32,187  (75.9) | 35,557  (72.1) |

AS: ankylosing spondylitis; CCAE: Commercial Claims and Encounters; PsA: psoriatic arthritis; RA: rheumatoid arthritis; rheum: rheumatologist exposure; yrs: years

**Supplementary Fig. S1** Study design


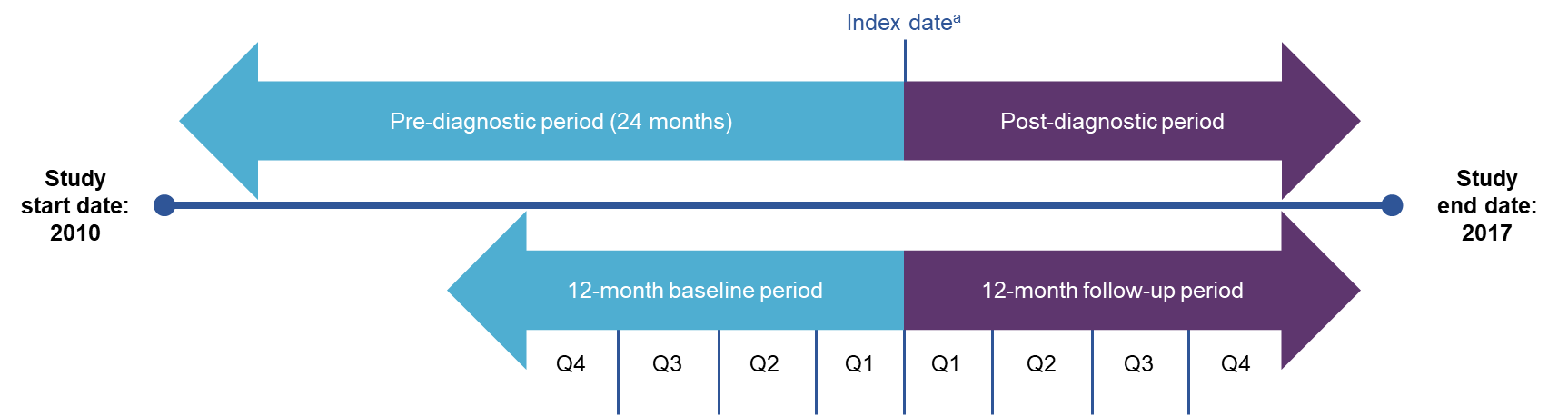


Patients were required to have ≥24 months preceding the incident diagnostic claim in which they were continuously enrolled and did not have a claim for the index disease. The 12 months directly preceding the index claim was used as the baseline period. The 12-month period of continuous enrollment following the index date was used as the follow-up period. Gaps of ≤60 days were allowed. ^a^Index date was defined as the date of the incident diagnostic claim for disease (patient-specific). Q: quarter

**Supplementary Fig. S2** Proportion of pharmacy claims for (**a**) NSAIDs and
(**b**) bDMARDs by quarter in baseline and follow-up for AS, PsA, and RA in CCAE and Medicaid populations


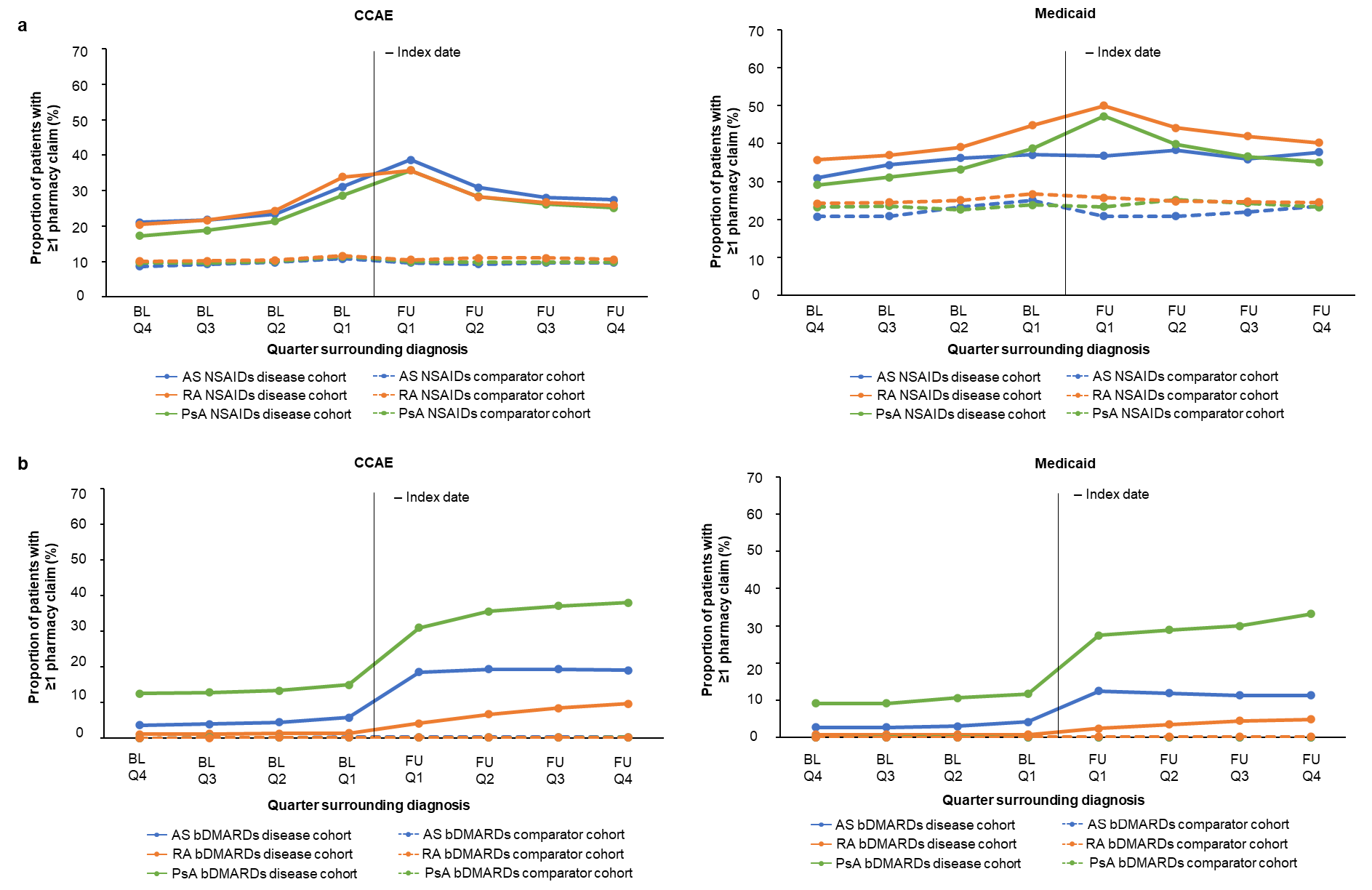


AS: ankylosing spondylitis; bDMARD: biologic disease‑modifying anti-rheumatic drug; BL: baseline; CCAE: Commercial Claims and Encounters; FU: follow-up; NSAID: non-steroidal anti‑inflammatory drug; PsA: psoriatic arthritis; Q: quarter; RA: rheumatoid arthritis

**Supplementary Fig. S3** Treatment exposures (**a**) and therapy combinations
(**b**) in follow‑up based on exposure to rheumatologist in baseline or day of diagnosis for patients with AS, PsA, and RA in CCAE population


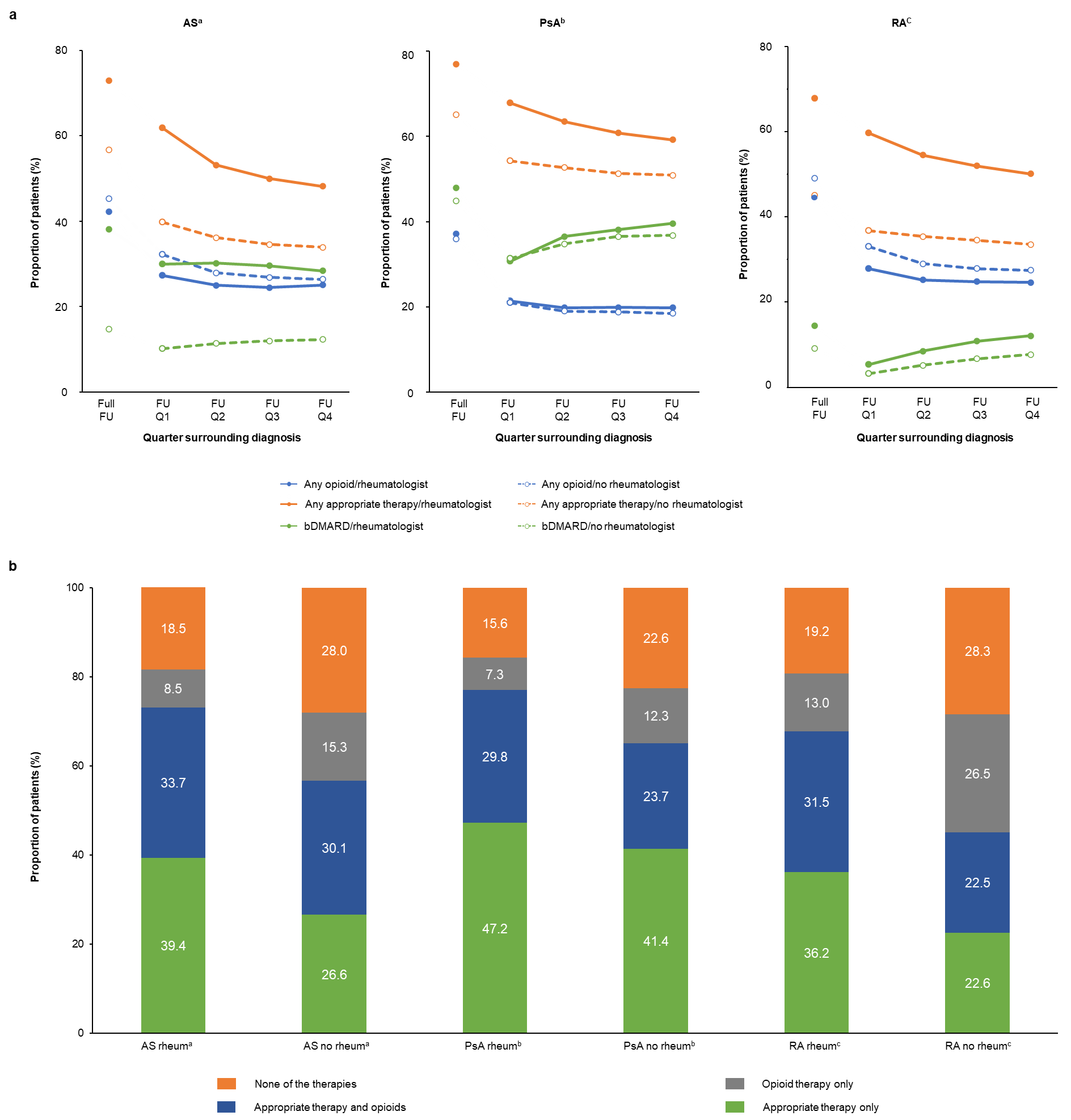


AS: ankylosing spondylitis; bDMARD: biologic disease-modifying antirheumatic drug; BL: baseline; CCAE: Commercial Claims and Encounters; FU: follow-up; PsA: psoriatic arthritis; Q: quarter; RA: rheumatoid arthritis; rheum: rheumatologist exposure.^a^2,444 AS patients had exposure to a rheumatologist, 3,325 did not; ^b^4,922 PsA patients had exposure to a rheumatologist, 5,958 did not; ^c^42,430 RA patients had exposure to a rheumatologist, 49,292 did not

**Supplementary Fig. S4** Proportion of disease appropriate therapy and opioids, alone or in combination, for disease by quarter in baseline and follow-up for AS, PsA, and RA in Medicaid population


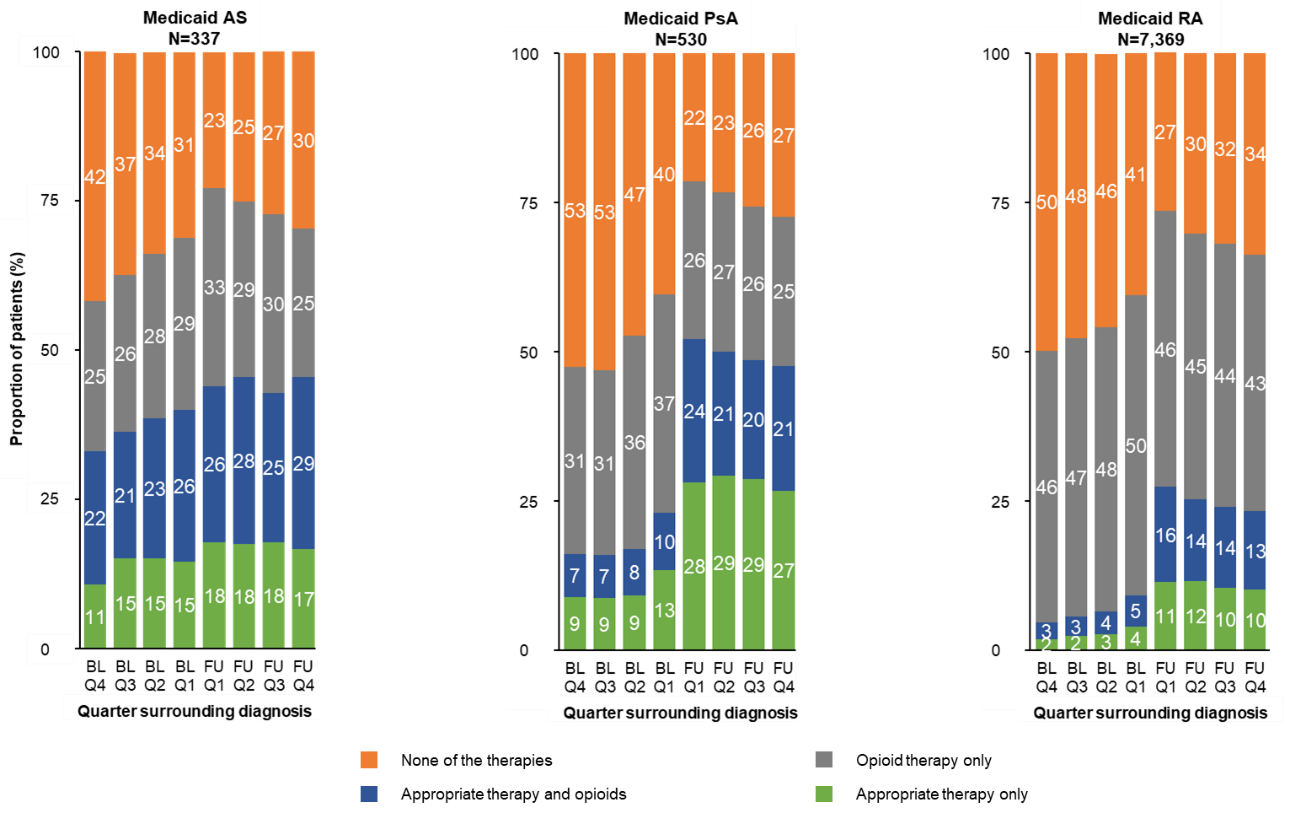


AS: ankylosing spondylitis; BL: baseline; FU: follow-up; PsA: psoriatic arthritis; RA: rheumatoid arthritis
